# Molecular Characteristics of Water-Insoluble Tin-Porphyrins for Designing the One-Photon-Induced Two-Electron Oxidation of Water in Artificial Photosynthesis

**DOI:** 10.3390/molecules28041882

**Published:** 2023-02-16

**Authors:** Arun Thomas, Yutaka Ohsaki, Ryosuke Nakazato, Fazalurahman Kuttassery, Siby Mathew, Sebastian Nybin Remello, Hiroshi Tachibana, Haruo Inoue

**Affiliations:** 1 Department of Chemistry, St. Stephen’s College, Uzhavoor P.O. Box 686634, Kerala, India; 2 Department of Applied Chemistry, Graduate School of Urban Environmental Sciences, Tokyo Metropolitan University, 1-1 Minami-Ohsawa Hachioji, Tokyo 192-0397, Japan; 3 Department of Chemistry, University of Calicut, Thenhipallam P.O. Box 673635, Kerala, India; 4 Department of Applied Chemistry, Cochin University of Science and Technology, Kochi P.O. Box 682022, Kerala, India

**Keywords:** artificial photosynthesis, molecular catalyst, tin-porphyrins, hydrogen peroxide, axial ligand, acid-base equilibrium

## Abstract

Faced with the new stage of water oxidation by molecular catalysts (MCs) in artificial photosynthesis to overcome the bottle neck issue, the “Photon-flux density problem of sunlight,” a two-electron oxidation process forming H_2_O_2_ in place of the conventional four-electron oxidation evolving O_2_ has attracted much attention. The molecular characteristics of tin(IV)-tetrapyridylporphyrin (SnTPyP), as one of the most promising MCs for the two-electron water oxidation, has been studied in detail. The protolytic equilibria among nine species of SnTPyP, with eight p*K*_a_ values on the axial ligands’ water molecules and peripheral pyridyl nitrogen atoms in both the ground and excited states, have been clarified through the measurements of UV-vis, fluorescence, ^1^H NMR, and dynamic fluorescence decay behaviour. The oxidation potentials in the Pourbaix diagram and spin densities by DFT calculation of the one-electron oxidized form of each nine species have predicted that the fully deprotonated species ([SnTPyP(O^−^)_2_]^2−^) and the singly deprotonated one ([SnTPyP(OH)(O^−^)]^−^) serve as the most favourable MCs for visible light-induced two-electron water oxidation when they are adsorbed on TiO_2_ for H_2_ formation or SnO_2_ for Z-scheme CO_2_ reduction in the molecular catalyst sensitized system of artificial photosynthesis.

## 1. Introduction

Among the various scientific approaches to obtaining a concrete grip on a sustainable society based on a carbon-neutral energy system [1,2,3], artificial photosynthesis with water as an electron donor to induce H_2_ evolution/CO_2_ fixation by sun light should be one of the most promising challenges to be developed [4,5,6,7]. Intensive efforts have been focused on hydrogen evolution by (1) electrochemical water splitting, utilizing photovoltaic electricity (PV/E) [8,9,10,11,12]; (2) semiconductor photocatalyst (SC) [13,14,15,16,17,18,19,20,21,22,23,24]; (3) molecular catalyst (MC) [25,26,27,28,29,30,31,32,33,34,35,36,37,38,39,40]; and (4) utilizing biological systems [41,42,43]. Although rather high energy conversion efficiencies have been reported in hydrogen evolution by the PV/E method [8,9,10,11,12], the two-step processes of creating electricity and the subsequent electrolysis inevitably force the PV/E method into the more severe condition of the renewable energy factor (REF: Energy output/Energy input >1) than the one-step process of the SC and MC methods [44]. The superior direct water splitting of SC methods, however, has the additional challenge of utilizing visible light [13,14,15,16,17,18,19,20]. The MCs can easily tune their structure to adapt the visible light absorption, while they suffer a serious “Photon-flux density problem” [44,45]. The extremely long time-interval of the next photon’s arrival (~0.17 s for Sn(IV)TPP [44]) under the rarefied light intensity of sun light almost supresses the multi-electron processes with the stepwise multi-photon absorption, such as the four-electron oxidation of water to evolve dioxygen due to the much faster charge recombination or decomposition/transformation of the oxidized molecular catalysts. To avoid the “Photon-flux density problem of sunlight” against the multi-photon processes, we have focused our efforts on developing a one-photon process of water oxidation that does not require waiting for the next photon’s arrival on the MCs. How the unfavorable side reactions of MCs can be avoided or minimized is indeed a crucial point in the MC methods. The one-photon-induced water oxidation methodology should be the answer to the problem, which is the central subject of this article. We have found the one-photon-induced epoxidation of alkene with water as both an electron and oxygen atom donor catalysed by metalloporphyrins [46,47,48,49,50,51,52,53,54,55]. We have further found the efficient electrochemical two-electron oxidation of water to form H_2_O_2_, which is initiated by the one-electron oxidation of Al- [56], Sn- [57,58], and Zn-porphyrins [59]. The photochemical two-electron water oxidation forming H_2_O_2_ has been attained by Ge-porphyrins [60] and the first exemplum of the simultaneous formation of H_2_/H_2_O_2_ by one-photon-induced water splitting has also been found in the molecular catalyst sensitized system (MCSS), composed of Al-porphyrins adsorbed on TiO_2_ nano particles [61,62,63]. The one-photon-induced two-electron water splitting into H_2_/H_2_O_2_ should be one of the most promising approaches to overcome the “Photon-flux density problem” and has superior points in both energy storage (∆*E* = 1.77 eV for H_2_/H_2_O_2_) and the spontaneous separation of H_2_ (gas) from H_2_O_2_ (liquid) to the conventional four-photon processes of water splitting into H_2_/O_2_ (∆*E* = 1.23 eV) that requires additional energy of separation. Now, the MCSS faces the next stage of development for a robust MCSS with high reactivity. The supramolecular approach has also been demonstrated to be valid for protecting the reactive species from side reactions, even in saline water [64]. Obtaining a deeper insight into the molecular characteristics under the actual conditions that MCs would experience in the water splitting processes is most requisite in an adequate designing of the MCs for artificial photosynthesis. Here, we will report the first detailed study on the molecular characteristics of water insoluble tin(IV)-tetrapyridylporphyrin (SnTPyP) as one of the promising molecular catalysts in MCSS for two-electron water splitting by visible light. The acid-base equilibria of the axial ligands of SnTPyP under various pH conditions in both the ground and excited states, the one-electron oxidation potentials, and the spin populations of the one-electron oxidized SnTPyP, as well as the energetics, have all been studied in detail, which provide crucial information in designing the MCSS.

## 2. Results and Discussion

### 2.1. Molecular Catalyst Sensitized System (MCSS)

**Scheme 1:**
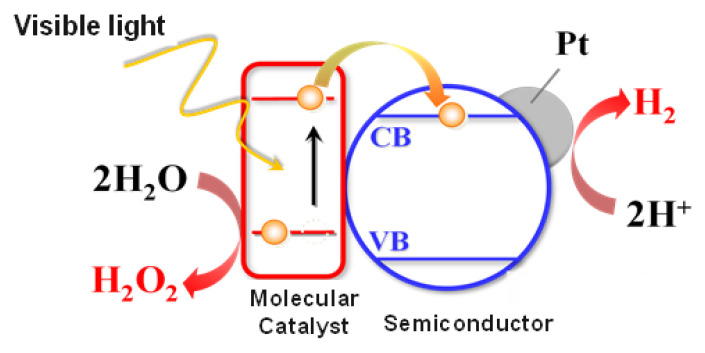
Molecular catalyst sensitized system for two-electron water splitting [41,42,43].

The MCSS composes of three components: a molecular catalyst (MC) absorbing visible light to drive water oxidation; a semiconductor (SC), such as TiO_2_ serving as a wire to transfer the injected electron from the excited MC adsorbed on SC to the reduction terminal; and a co-catalyst, such as the Pt driving the hydrogen evolution (Scheme 1) [41,42,43].

In designing the MCSS to develop a high reactivity of two-electron water splitting into H_2_/H_2_O_2_, in addition to some other factors, deeper insights into the following points are required: (1) How do the MCs efficiently absorb sun light? (2) How are the MCs adsorbed on the surface of SC? (3) What are the microscopic orientations between the molecular orbitals between the MC and conduction band of the SC to enhance the electron injection? (4) How exergonic are the electron injections from the excited MCs into the conduction band of the SC? (5) How reactive are the one-electron oxidized forms of MCs against water/OH^−^? (6) How stable is the adsorption of MCs on the surface of the SC against variable actual conditions during the reactions? (7) What are the reactivities of the hydrogen evolution on the co-catalysts? (8) How could the monomeric dispersion of the SC be maintained without aggregation/agglomeration in the water to assure the constant pH conditions surrounding the MCs?

**Scheme 2:**
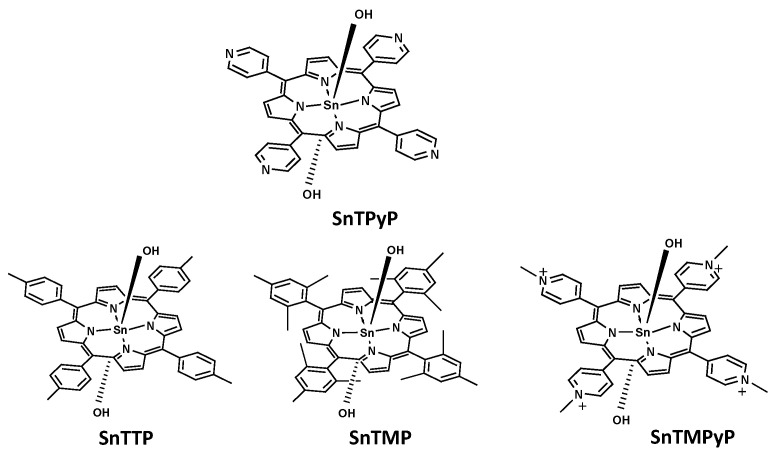
Molecular structure of water-insoluble tin(IV)-porphyrins (SnTPyP, SnTTP, SnTMP) and water-soluble one (SnTMPyP).

In regard to point (1), SnTPyP has a strong absorptivity against visible light (ε = 6.36 × 10^5^ M^−1^ dm^−3^ (λ_max_: 418 nm in MeOH), 2.15 × 10^4^ M^−1^ dm^−3^ (λ_max_: 553 nm in MeOH)) to satisfy the criteria, as do other metalloporphyrins [48,49,50,51,52,53,54,55]. Ionic adsorptions of water-soluble MCs on the surface of SC have been frequently adopted, while they are rather unstable to induce unfavorable desorption under the fluctuating microscopic pH conditions surrounding the MCs in the gap space of the SC particles (point 2, 3, 8). Another mode of covalent adsorption through the coordination of the axial ligand, −OH, on the metal center of porphyrins would be one of the promising methodologies [62] to avoid the unfavorable desorption, where water-insoluble SnTPyP would be expected not to swim over the water phase but to remain on the surface of the SC. In these contexts, here, the molecular characteristics of water-insoluble *trans*-dihydroxy-5,10,15,20-tetra(4′-pyridyl)porphyrinato tin (IV) (SnTPyP) as an axially coordinative MC were studied for the first time in detail on the points (4) and (5) to compare with other water-insoluble tin-porphyrins (Scheme 2), such as 5,10,15,20-tetra(4′-tolyl)porphyrinato tin (IV) (SnTTP), 5,10,15,20-tetramesitylporphyrinato tin (IV) (SnTMP), and water-soluble 5,10,15,20-tetra(4′-methylpyridiniumyl)porphyrinato tin (IV) (SnTMPyP) [57,58].

### 2.2. Acid-Base Equilibria of Axial Ligands Water of SnTPyP in the Ground State

**Scheme 3:**
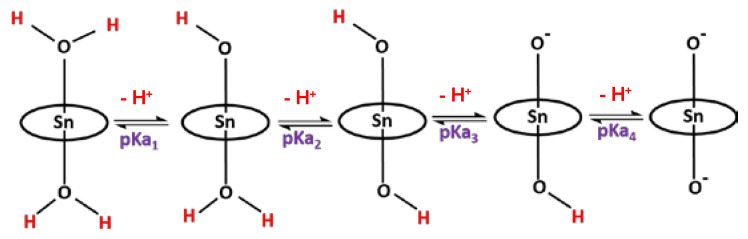
Protolytic equilibria of axial ligands of Sn-porphyrins.

The axial ligands, water, on the metal centre of metalloporphyrins are known to suffer stepwise protolysis under various pH conditions [56,57,58,59]. In designing a MCSS, specifying which species is photochemically excited under the reaction conditions should be one of the most necessary pieces of information. The SnTPyP examined here has two OH groups in the axial ligands to be [Sn(IV)TPyP(OH)_2_] as prepared (SI-S1, Supplementary Materials) [65,66]. To observe the protolysis of the axial ligands, the water-insoluble SnTPyP was once solubilized into an organic solvent, such as acetonitrile (CH_3_CN), and subsequently, water with various pH values was added. In the cases of other water-insoluble SnTTP and SnTMP, as a typical example, the *λ_max_s* of Soret bands at around 420 nm of the UV-vis spectrum in CH_3_CN/H_2_O (9/1, *v*/*v*) monotonically blue-shifted when the pH values decreased, exhibiting a four-step change with four isosbestic points in each step to indicate the presence of four p*K*_a_ values for stepwise protolysis among five species, as depicted in Scheme 3 [57,58].

**Figure 1:**
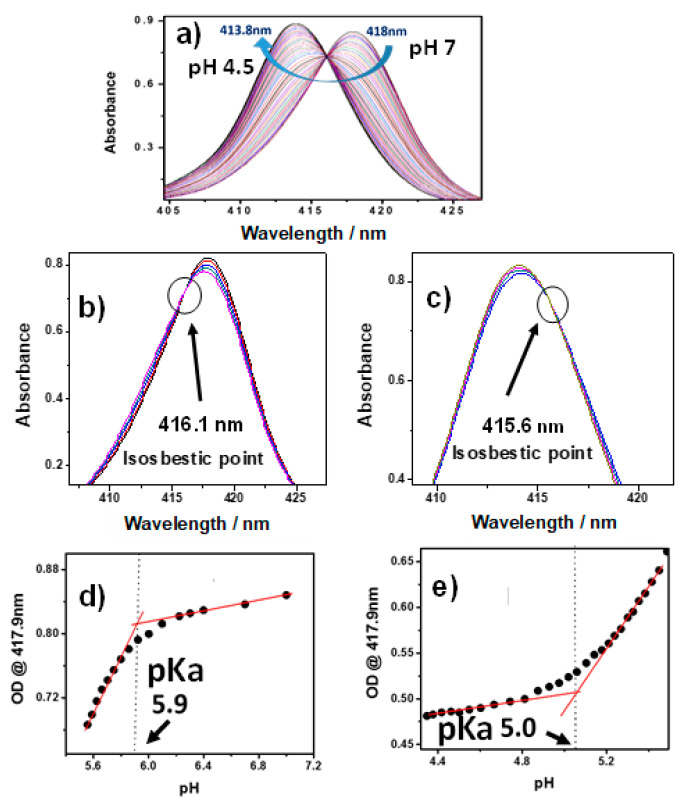
(**a**) Blue shifting of the *λ_max_* (Soret band) of SnTPyP (1.4 × 10^−6^ M in CH_3_CN/H_2_O (8/2, *v*/*v*)) in the pH region 7 to 4.5, (**b**) isosbestic point (circle) observed in the pH region 7–5.5, (**c**) isosbestic point (circle) observed in the pH region 5.5–4.2, (**d**,**e**) p*K*_a_ value estimated from the inflection point in the plots between pH vs. OD at 417.9 nm in the pH region 7–4.2.

SnTPyP, however, showed more complicated protolytic behavior. In contrast to SnTTP and SnTMP, the four pyridyl nitrogen atoms would possibly become protonated, in addition to the protolysis of the axial groups. Initially, the *λ_max_* (418 nm) of the absorption spectrum exhibited two-step blue shifting to 413.8 nm, with two isosbestic points at 416.1 and 415.6 nm, when the pH decreased from 7 to 4.5 (Figure 1a–c). The plot of the absorbance against the pH value showed two clear inflection points, reflecting two p*K*_a_ values, 5.9 and 5.0 (Figure 1d,e).

**Figure 2:**
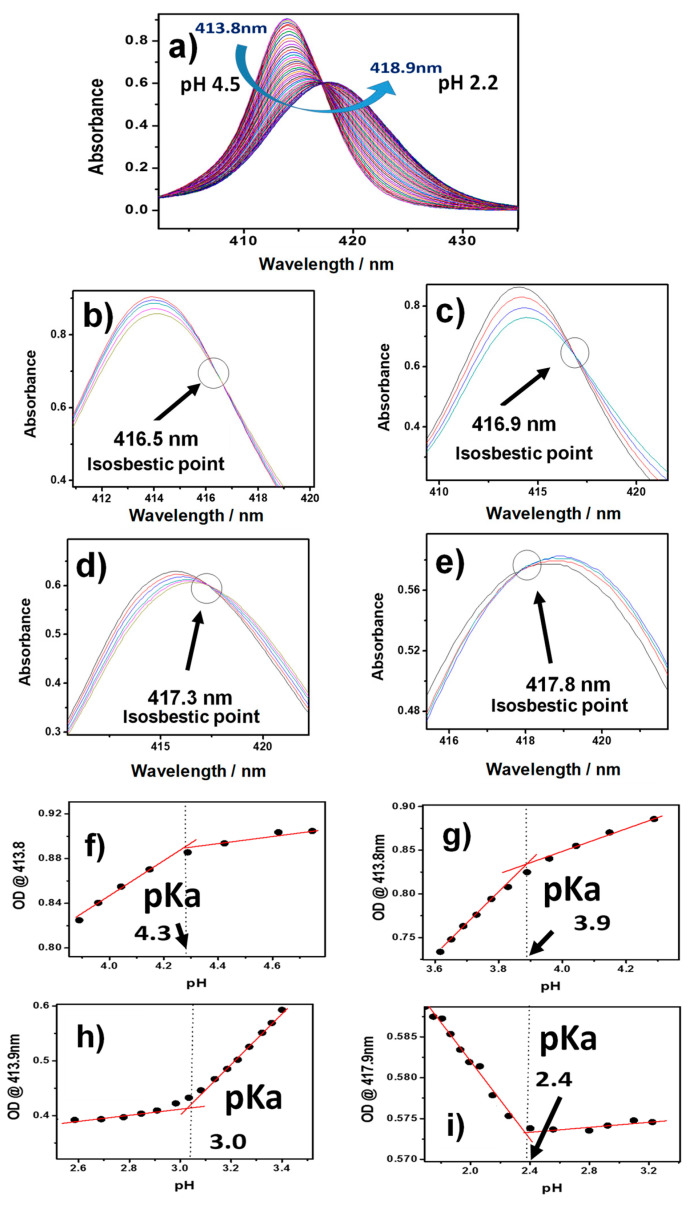
(**a**) Opposite red shifting of the *λ_max_* (Soret band) of SnTPyP (1.4 × 10^−6^ M in CH_3_CN/H_2_O (8/2, *v*/*v*)) in the pH region 4.5 to 2.2, (**b**–**e**) isosbestic points (circles) observed in the pH region 4.5–2.2, (**f**–**i**) p*K*_a_ values estimated from the inflection points in the plots between pH vs. OD in the pH region 4.5–2.2.

In contrast to the blue-shifting (Figure 1a), when the pH of the solution further being decreased from pH 4.5 to pH 2.2, a four-step red shift was observed (Figure 2a), with four isosbestic points at 416.5, 416.9, 417.3, 417.8 nm (Figure 2b–e). Each inflection point observed in the plot between the absorbance and the pH value indicates the presence of four p*K*_a_ values of 4.3, 3.9, 3.0, and 2.4, respectively (Figure 2f–i).

**Figure 3:**
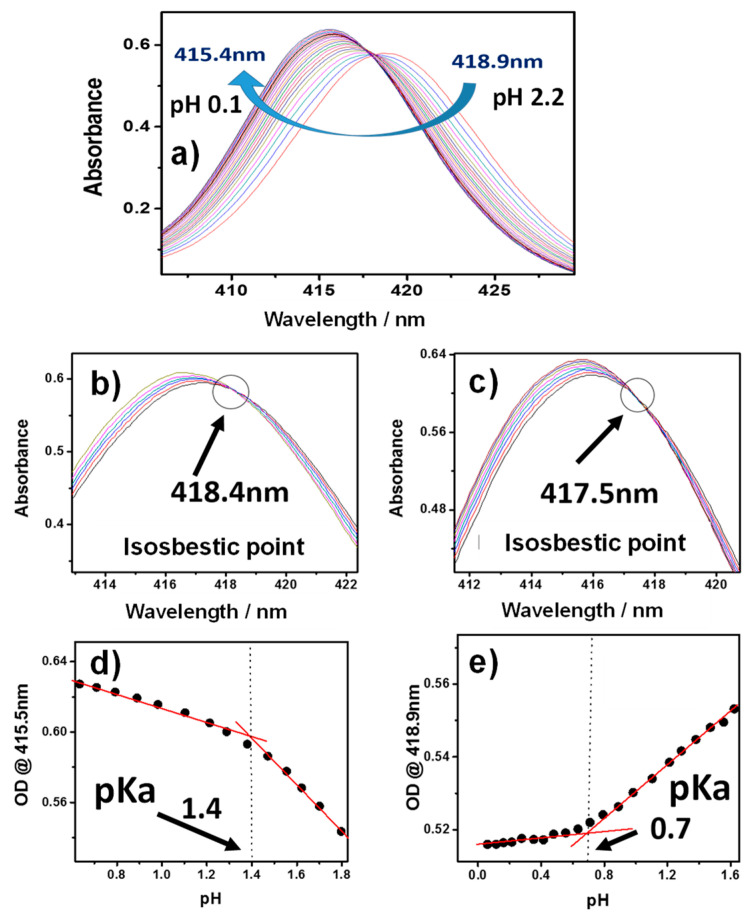
(**a**) The second blue shifting of the *λ_max_* (Soret band) of SnTPyP (1.4 × 10^−6^ M in CH_3_CN/H_2_O (8/2, *v*/*v*)) in the pH region 2.2–0.4, (**b**,**c**) isosbestic points (circle) observed in the pH region 2.2–0.4, (**d**,**e**) pKa values estimated from the inflection points in the plots between pH vs. OD in the pH region 2.2–0.4.

Very interestingly, a further decrease in the pH value from 2.2 to 0.1 induced, again, an obvious two-step blue shift of the *λ_max_* from 418.9 nm to 415.4 nm (Figure 3a), with two isosbestic points at 418.4 and 417.5 nm in an opposite direction than the previous pH region from 4.5 to 2.2 (Figure 3b,c). Two p*K*_a_ values of 1.4 and 0.7 were also found in the plot of Figure 3d,e.

The UV-vis spectral changes under the various pH conditions were similarly observed in the fluorescence spectra (Figure S1a–d, Supplementary Materials). SnTPyP shows two characteristic emission bands at 596 and 650 nm under the neutral condition when excited at 415 nm. These bands each exhibited a blue shift to 592 nm and 646 nm in a multi-step manner when the pH value changed from 7 to 0.1 (Figure S1a). The p*K*_a_ values were estimated from the inflection points in the plots of the pH vs. fluorescence intensity at the fixed wavelength to be 6.0, 5.0, 4.4, 3.8, 2.9, 2.5, 1.5, and 0.6, which are well-matched with the p*K*_a_ values observed by the UV-vis spectrophotometric titration, as shown above.

The eight p*K*_a_ values (5.9 (6.0), 5.0 (5.0), 4.3 (4.4), 3.9 (3.8), 3.0 (2.9), 2.4 (2.5), 1.4 (1.5), and 0.7 (0.6)) observed in the UV-vis spectral changes and the fluorescence changes (in parenthesis), indicate the presence of an acid-base equilibria among nine protolytic species. At least the five protolytic species in respect to the axial ligands, water, shown in Scheme 3 should be involved in the nine species. The other four species could be assigned as protolytic species on the peripheral pyridine groups, as anticipated above. To inspect the presumption, the ^1^H NMR spectrum under the various pH conditions was further examined. In the case of SnTTP with two axial ligands, water, but without pyridyl nitrogen atoms on the peripheral substituents of the porphyrin ring, a typical AB splitting peak pattern is observed for the *ortho*- (*δ*~8.22 ppm) and *meta*-protons (*δ*~7.71 ppm) of the peripheral tolyl groups in its ^1^H NMR under the neutral condition (Figure S2a). When the pH of the solution decreased, the chemical shifts of the tolyl protons almost remained unchanged, while a slight broadening was observed below pH 2. Contrasting to the silence of the tolyl protons against pH, the pyrrole protons (*δ*~9.17 ppm under the neutral condition) suffered gradual downfield shifting to *δ*~9.28 ppm at pH 5 and down to *δ*~9.41 ppm at pH 1. These indicate that the magnetic environment for the pyrrole protons on the porphyrin ring is directly affected by the electron density of the two axial ligands varied by their protolysis. The protons on the phenyl substituents, however, are rather insensitive to the change of the axial ligands, mostly due to a limited conjugation between the porphyrin ring and the twisted phenyl substituents. Viewing the effect of the protolytic behavior of the axial ligands, the ^1^H NMR of SnTPyP was measured here in CD_3_CN/D_2_O (8/2, *v*/*v*) under various pH conditions.

**Figure 4:**
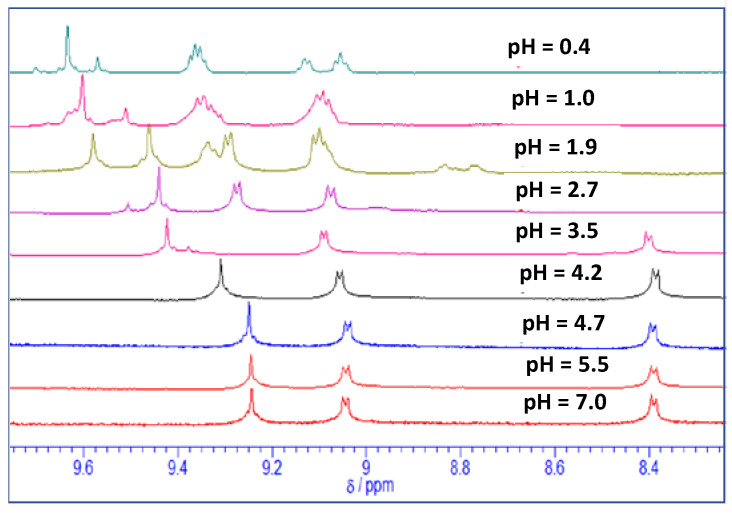
^1^H NMR of SnTPyP in CD_3_CN/D_2_O (8/2, *v*/*v*) under various pH conditions. The pH was adjusted by adding deuterated sulfuric acid.

As shown in Figure 4, by changing the pH from 7 to 4.7, where the substantial blue shift of *λ_max_* was observed in the UV-vis spectra (Figure 1a), the *β*-pyrrolic protons (*δ*~9.24 ppm) exhibited a slight shift to *δ*~9.25 ppm at pH 4.7, while the *ortho*- (*δ*~8.38 ppm) and *meta*-protons (*δ*~9.04 ppm) remained almost unchanged in their ^1^H NMR. The p*K*_a_ values 5.9 and 5.0 could thus be assigned to the protolysis of the axial ligands (Equations (1) and (2)).

[SnTPyP(O^−^)_2_]^2−^ + H^+^ ⇌ [SnTPyP(O^−^)(OH)]^−^        (p*K_a_* = 5.9) (1)

[SnTPyP(O^−^)(OH)]^−^ + H^+^ ⇌ [SnTPyP(OH)_2_]        (p*K_a_* = 5.0) (2)

Then, in the region of pH 4.2–3.5, where the opposite red shift of *λ_max_* was observed in the UV-vis spectra (Figure 2a), the ^1^H NMR showed downfield shifts of both *β*-pyrrolic (*δ*~9.425 at pH 3.5) and *meta*-protons (*δ*~9.09 at pH 3.5) due to the protonation to the peripheral pyridyl nitrogen atoms. At pH 2.7, all the peaks shifted largely to the downfield (ortho- *δ*~9.075, *meta*- *δ*~9.275, and *β*-pyrrolic- *δ*~9.44, 9.50 (minor)). The substantial downfield shift of the pyridyl protons indicates the protonation to the pyridyl nitrogen atoms, as observed in the SnTMPyP with pyridinium substituents (ortho- *δ*~9.025, *meta*- *δ*~9.320, and *β*-pyrrolic- *δ*~9.39 at pH 7 (Figure S2b)). The four p*K_a_*s (4.3, 3.9, 3.0, and 2.4) are thus assigned to those of Equations (3)–(6), as follows.

[SnTPyP(OH)_2_] + H^+^ ⇌ [SnT(PyH^+^)P (OH)_2_]^+^                   (p*K_a_* = 4.3) (3)

[SnT(PyH^+^)P (OH)_2_]^+^ + H^+^ ⇌ [SnT(PyH_2_^2+^)P (OH)_2_]^2+^    (p*K_a_* = 3.9) (4)

[SnT(PyH_2_^2+^)P (OH)_2_]^2+^ + H^+^ ⇌ [SnT(PyH_3_^3+^)P(OH)_2_]^3+^  (p*K_a_* = 3.0) (5)

[SnT(PyH_4_^3+^)P (OH)_2_]^3+^ + H^+^ ⇌ [SnT(PyH_4_^4+^)P(OH)_2_]^4+^  (p*K_a_* = 2.4) (6)

A further decrease in the pH 1.9–0.4, where the second blue shift of *λ_max_* was observed in the UV-vis spectra (Figure 3a), induced a further downfield chemical shift (Figure 4) for the *β*-pyrrolic-protons (*δ*~9.63 at pH 0.4), while the chemical shifts of the *ortho*- and *meta*-protons of the protonated pyridinium groups were less affected. The rest of the two p*K*_a_ values (1.4 and 0.7) could thus be ascribed to the protolysis of the axial ligands in Equations (7) and (8).

[SnT(PyH_4_^4+^)P (OH)_2_]^4+^ + H^+^ ⇌ [SnT(PyH_4_^4+^)P(OH)(H_2_O)]^5+^  (p*K_a_* = 1.4) (7)

[SnT(PyH_4_^4+^)P (OH)(H_2_O)]^5+^ + H^+^ ⇌ [SnT(PyH_4_^4+^)P(H_2_O)_2_]^6+^ (p*K_a_* = 0.7) (8)

**Scheme 4:**
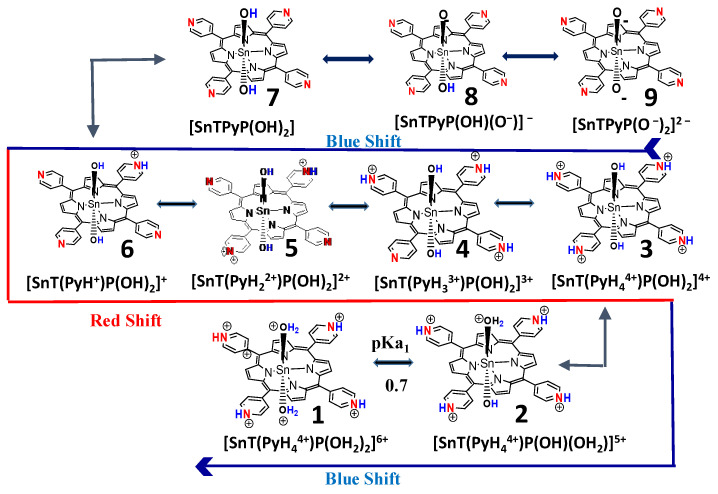
Acid-base equilibria among nine protolytic species of SnTPyP in respect to the axial ligand water molecules and the peripheral pyridyl groups.

The acid-base equilibria among the nine species with eight p*K*_a_ values are summarized as Scheme 4. The fully protonated species, [SnT(PyH_4_^4+^)P(H_2_O)_2_]^6+^, is numbered as **1**; the fully deprotonated one, [SnTPyP(OH)_2_], to be **9**; and the remaining species were numbered in order.

A simple question may arise here in the stepwise protonation with four p*K*_a_ to the pyridyl nitrogen atoms. When a proton attaches to the pyridyl nitrogen, the four nitrogen atoms should equally face the proton. Why do the four protons not attach simultaneously with one p*K*_a_ to the four pyridyl groups? To obtain a deeper insight into this point, a DFT calculation was carried out for the protonation processes. The electron density of each pyridyl nitrogen atom was calculated as a natural bond orbital population analysis charge (NBO charge) [67,68]. Supposing that a single proton attaches to one of the four pyridyl nitrogen atoms, the electron density on each of the three free nitrogen atoms is varied, as shown in Figure S3a–d, Supplementary Materials. Interestingly, upon a single-protonation to one of the pyridyl nitrogen, the NBO charge of the other three free pyridyl nitrogen atoms becomes smaller (∆(NBO charge) = +0.0012 to +0.00145); that is, the electron densities of the three free pyridyl nitrogen atoms all become lower than the starting four free nitrogen atoms, which rationalize the single-protonation (Figure S3a). The further protonation to the rest of the free pyridyl nitrogen atoms could thus not proceed under the same pH condition for the first protonation. Similarly, the second protonation (double-protonation) to the singly-protonated species also induces the lower electron densities of the two free pyridyl nitrogen atoms (∆(NBO charge) = +0.00106 to +0.00145) than those of the three free ones of the singly-protonated species (Figure S3b). In addition, the free pyridyl nitrogen atom in the triply-protonated species has a lower electron density (∆(NBO charge) = +0.00113) than those in the doubly-protonated one (Figure S3c). The lowering of the electron densities of the rest of the free pyridyl nitrogen atoms upon each protonation step therefore rationalizes the stepwise protonation processes. In regard to the doubly protonated forms, there should be two isomers of *cis*- and *trans*-form. The DFT calculation also predicts that the *trans*-form is slightly more stable than the *cis*-form, by 0.12 kJ/mol, which indicates almost an equal population of *trans*-form/*cis*-form (51/49) at 300 K; that is, the two protons would be hopping among the four pyridyl groups. The eight p*K*_a_ values among the nine species provide crucial information about which species is photochemically excited under the actual pH condition given for the actual reaction system.

### 2.3. Acid-Base Equilibria in the Excited State

**Figure 5:**
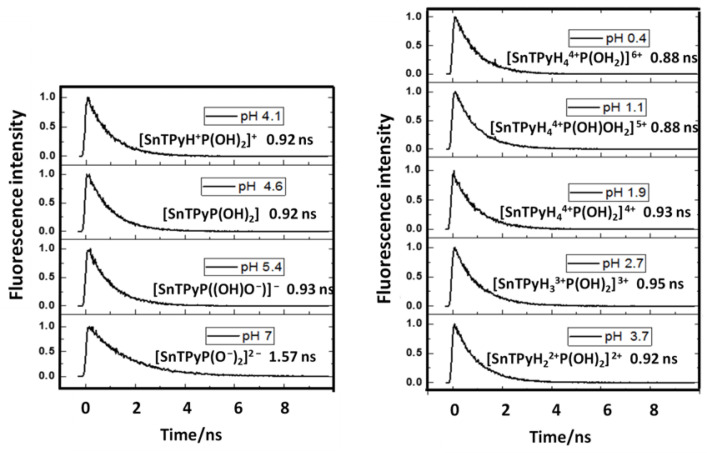
Fluorescence decay profile of each excited SnTPyP in different protolytic form on the axial ligands and peripheral pyridyl groups.

The photochemistry of the MC starts from an electronic excitation of the MC in the ground state and a primary step of photoreaction begins at the corresponding excited state. Then, it is also crucial to obtain insight into the condition of the acid-base equilibria in the excited state. Are the excited nine species dynamically equilibrated within their lifetimes or do they remain at their own state without mutual interconversion? To examine the detail on the points, the fluorescence dynamic decay behavior was observed by a single-photon-counting condition using a pico second laser pulse (see Materials and Methods). As shown in Figure 5, each of the nine species was selectively excited by varying the pH condition based on the eight p*K*_a_ values (Scheme 4). The longest lifetime was 1.57 ns for the fully deprotonated excited species (^1^[SnTPyP(O^−^)_2_]^2−^* (9*)) and the singly protonated one (^1^[SnTPyP(OH)(O^−^)]^−^* (8*)) has a substantially shorter lifetime of 0.93 ns with single exponential decays for both cases, indicating that the two adjacent protolytic species are not dynamically equilibrated to remain in their locally excited states corresponding to their ground states, respectively. The other seven excited protolytic species all exhibited single exponential decays, with various lifetimes of 0.95–0.88 ns, also indicating no dynamic equilibration in their excited states. The primary processes from their excited states should thus start from each local excited species corresponding to the ground state given under the actual pH condition of the reaction system.

### 2.4. One-Electron Oxidation of SnTPyP

**Figure 6:**
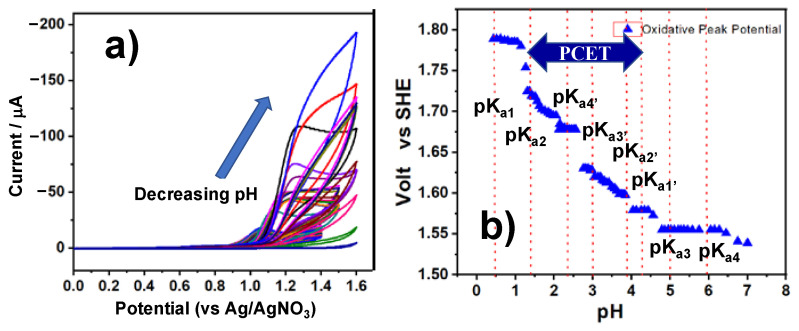
(**a**) Cyclic voltammogram of SnTPyP (0.2 mM in CH_3_CN/H_2_O (8/2, *v*/*v*)) in the presence of (C_4_H_9_N)_4_PF_6_ (0.1 M) as an electrolyte with boron-doped diamond as the working electrode, Pt coil as the counter electrode, and Ag/AgNO_3_ as the reference electrode, scan rate (100 mV/s), (**b**) Pourbaix diagram of SnTPyP under various pH conditions.

In designing a water oxidation system of MCSS in an artificial photosynthesis, where a light absorbing MC adsorbed on a semiconductor is expected to inject an electron to start the photochemical anodic cycle, an energetic relation of how feasible the electron injection from the excited MC into the conduction bands of semiconductors should be the key information. To obtain insight into this point, the one-electron oxidation potentials of SnTPyP were measured through the cyclic voltammetry (CV). As shown in Figure 6a, the CVs of the SnTPyP under various pH conditions all exhibited irreversible catalytic oxidation waves, indicating that the first oxidation waves reflect the catalytic water oxidation. From pH 7 to 4.5 in the Pourbaix diagram (Figure 6b), where the first blue shift was observed in the UV-vis spectra (Figure 1a), there is no significant difference in the oxidation potential. On the other hand, from pH 4.5 to pH 2, where the opposite red shift in the UV-vis spectra (Figure 2a) and the large downfield shift in the NMR (Figure 4) were induced, the potential linearly increased with the decrease in the pH value (∆*E*/∆pH = −0.056 V). The pH dependency almost corresponds to a theoretical value (0.059 Volt) predicted by Nernst equation, indicating a proton coupled electron transfer process. In the region lower than pH 1, the oxidation potential again remained unchanged, while the current decreased gradually due to the phase separation. In the proton coupled process in the region pH 4.5–2, the oxidation potential of SnTPyP was expressed as *E_ox_* (Volt vs. SHE) = −0.056 × pH + 1.82 by the linear relation in the Pourbaix diagram (Figure 6b). Which proton is liberated upon the one-electron oxidation in the pH region of 4.5–2? Either the proton on the axial ligand or the pyridinium group could dissociate. The DFT calculation clearly predicts that the pyridinium proton is more labile than that of the axial ligand OH group in every one-electron oxidation step of each protonated species, such as Equations (9)–(12), respectively.

[SnT(PyH^+^)P(OH)_2_]^+^ − e^−^ − H^+^~ ∆*E*([SnTPyP(OH)_2_]^+•^ − [SnT(PyPH^+^)(OH)(O^•^)]^+•^) = −19.0 kcal/mol (9)

[SnT(PyH_2_^+^)P(OH)_2_]^2+^ − e^−^ − H^+^~ ∆*E*([SnT(PyH^+^)P(OH)_2_]^2+•^ − [SnT(PyH_2_^+^)P(OH)(O^•^)]^2+•^) = −16.2 kcal/mol (10)

[SnT(PyH_3_^+^)P(OH)_2_]^3+^ – e^−^ – H^+^ ~ ∆*E*([SnT(PyH_2_^+^)P(OH)_2_]^3+•^ − [SnT(PyH_3_^+^)P(OH)(O^•^)]^3+•^) = −13.3 kcal/mol (11)

[SnT(PyH_4_^+^)P(OH)_2_]^4+^ − e^−^ − H^+^~ ∆*E*([SnT(PyH_3_^+^)P(OH)_2_]^4+•^ − [SnT(PyH_4_^+^)P(OH)(O^•^)]^4+•^) = −10.4 kcal/mol (12)

In designing a MCSS using a typical *n*-type semiconductor, such as TiO_2_ or SnO_2_, as an electron wire (Scheme 1), the energetics of the electron injection from the excited SnTPyP adsorbed on the semiconductor into the conduction band of TiO_2_ or SnO_2_ would also be crucial information. The *n*-type semiconductor, TiO_2_, could serve as the electron wire to the reduction terminal end for H_2_ formation [53,61], and SnO_2_ does this for CO_2_ reduction through Z-scheme artificial photosynthesis [29,30,31,32,33]. The energy required for the electron injection was calculated from the excitation energy (0-0 band) of SnTPyP (~2.1 eV), the oxidation potential of SnTPyP, and the conduction band energy of TiO_2_ or SnO_2_ under various pH conditions, as summarized in Table 1. According to the Nernst equation, *E*_cb_ is dependent upon the pH as the shifting to the negative direction by 59 mV/pH with the increase in the pH value, while *E*_ox_ of SnTPyP (for **1**, **2**, **7**–**9** except **3**–**6**), are independent of the pH, as seen in Table 1, and the excited energy is also independent of the pH. Interestingly, almost all the cases of the electron injection from the excited ^1^SnTPyP* into the conduction band of TiO_2_ or SnO_2_ have feasible negative ∆*E* (−0.09 to −0.32 eV), while only the excited state of the fully deprotonated species, [SnTPyP(O^−^)_2_]^2−^ (**9**), might be questionable for inducing an efficient electron injection into the conduction band of TiO_2_ because of the rather small ∆*E* = −0.01 eV).

### 2.5. Spin Density of the One-Electron Oxidized Species

In the two-electron water oxidation to form H_2_O_2_, a nucleophilic attack of OH^−^/H_2_O to the activated axial ligand oxygen atom upon the one-electron oxidation of the starting catalyst, metalloporphyrins, is the key process of the reaction cycle [56,57,58,59,60,61,62,63,64]. When the electron spin of the one-electron oxidized species in its doublet state is localized on the axial oxygen ligand, the DFT calculation predicts that the nucleophilic attack of OH^−^/H_2_O is most preferable for the H_2_O_2_ formation, and it was demonstrated by a transient laser flash photolysis study in the case of AlTMPyP on TiO_2_ [62]. On the other hand, a small population of the electron spin on the axial oxygen ligand with a substantial delocalization of the porphyrin ring leads to an extremely low reactivity for the nucleophilic attack [56]. For the two-electron water oxidation, the reactivity is thus mostly governed by the oxyl-radical character of the axial ligand OH group on the MCs, which can be predicted by the electron spin density on the axial ligand. The higher spin density would indicate the higher reactivity of the two-electron water oxidation; that is, the avoiding/minimizing unfavorable side reactions would be expected to the more extreme. Thus, in designing an MCSS, the spin density of the one-electron oxidized species formed by an electron injection from the excited MCs would be a good measure to provide key information for the system to be developed. In Table 1, the spin density on each axial oxygen ligand predicted by the DFT calculation is tabulated, as well as being visualized in Figure S4, Supplementary Materials. Interestingly, only the two species among the nine one-electron oxidized forms, the fully deprotonated one ([SnTPyP(O^−^)(O^•^)]^−•^) and the second fully deprotonated one ([SnTPyP(OH)(O^•^)]^•^, have their electron spins exclusively on one of the axial oxygen ligands (spin density: 0.988, 0.993); namely, the axial oxygen ligands are activated as oxyl radicals, which are very reactive against OH^−^/H_2_O_2_ to form hydroperoxy intermediates. The other seven species, existing under the conditions of pH < 5, have their electron spins all delocalized on the porphyrin rings (Figure S4), indicating that they are less reactive against H_2_O_2_ formation, even when the electron injection into the conduction band of TiO_2_ or SnO_2_ is feasible (Table 1). It turns out that the one-electron oxidized form of [SnTPyP(O^−^)_2_]^2−^ have the most preferable reactivity against two-electron water oxidation, but has insufficient energy against the electron injection into the conduction band of TiO_2_, which is in contrast to the case for SnO_2_. This is a conclusive prediction by this study. The fully deprotonated species shall thus be adopted for SnO_2_ system.

## 3. Materials and Methods

### 3.1. Materials

*trans*-Dihydroxy-5,10,15,20-tetra(4′-pyridyl)porphyrinato tin (IV) (SnTPyP) was synthesized by the reaction between SnCl_2_ and free base H_2_TPyP in water at ambient temperature with good yield (93%), according to the previous report (SI-S1, Supplementary Materials) [65,66]. Free base 5,10,15,20-tetra(4′-pyridyl)porphyrin (H_2_TPyP), acetonitrile (CH_3_CN), and SnCl_2_ were purchased from TCI chemicals Co., Ltd., Tokyo, Japan. All the common laboratory solvents and general chemicals used for present study were purchased from Kanto Chemicals Co., Inc., (Tokyo, Japan) and used without further purifications. Deionized water was prepared by passing through an ion-exchange column (G-10, Organo Co., Tokyo, Japan), while maintaining the conductance of water below 0.1 μScm^−1^. ^1^H NMR solvents such as CD_3_CN, D_2_O (Kanto Chemicals Co., Inc. Tokyo, Japan), NaOD, and D_2_SO_4_ (Sigma Aldrich, Tokyo, Japan) were used as received.

### 3.2. Measurements

The ^1^H NMR spectra in CD_3_CN/D_2_O (8/2, *v*/*v*) were measured on a BRUKER-500 MHz. The steady state absorption spectra were recorded on a Shimadzu UV-2550 spectrophotometer. The steady state fluorescence spectra were recorded on a JASCO FP-6500 spectrofluorometer. The fluorescence lifetime of SnTPyP in a nanosecond time scale were measured using a Nd^3+^ YAG laser-pumped OPG (EKSPLA, PL2143B + PG401; FWHM 25 ps, 5 Hz) for the excitation under the single photon counting condition. Fluorescence decay was monitored by a streak camera (Hamamatsu, C4334) equipped with polychromator (CHROMEX, 250IS). All the decay curves were analyzed using Igor pro 6.34 (A) software. Electrochemical experiments were conducted using an electrochemical analyzer (ALS 611DST, ALS Co., Ltd., Tokyo, Japan) at 25 °C. Cyclic voltammograms were measured in a three-component cell equipped with a glassy carbon electrode or a boron doped diamond electrode (BDD: 10,000 ppm) [69] as the working electrode, Ag/AgNO_3_ as the reference electrode, and Pt coil as the counter electrode. All the potentials were corrected to standard hydrogen electrode (SHE) by the addition of 0.539 V (Ag/AgNO_3_) to the measured potentials. The pHs of the sample solutions were adjusted by sulfuric acid and standard sodium hydroxide solution (Wako chemicals, Osaka, Japan) and measured by a pH meter (SK-620 pH meter, SATO Keiryoki mfg. Co., Ltd., Tokyo, Japan).

### 3.3. Density Functional Theory (DFT) Calculations

The Gaussian 16 program [70] was used in the calculations. The geometry optimization was carried out through the DFT method, with the use of the B3LYP function and the basis set of 6-31G(d) under the polar environment (PCM) of methanol as the solvent.

## 4. Conclusions

The molecular characteristics of water-insoluble tin-porphyrin, 5,10,15,20-tetra(4′-pyridyl)porphyrinato tin (IV) (SnTPyP), as a promising molecular catalyst for constructing a molecular catalyst sensitized system of an artificial photosynthesis were studied in detail. The acid-base equilibria among nine species in both the ground and excited states were clarified by UV-vis, ^1^H NMR, and dynamic fluorescence spectral measurements under various pH conditions. An irreversible catalytic anodic current was observed in the CV measurements in aqueous acetonitrile solution and the Pourbaix diagram was made from the oxidation potential for each nine species at the corresponding pH conditions. The energetics of the electron injection from the excited SnTPyP into the conduction band of a *n*-type semiconductor, such as TiO_2_ for the H_2_ evolving terminal or SnO_2_ for the Z-scheme CO_2_ reduction, estimated that all eight species of SnTPyP have the sufficient exergonic requirement, except nearly the equiergonic case of the excited state of fully deprotonated one ([SnTPyP(O^−^)_2_]^2−^), into TiO_2_. The theoretical prediction by the DFT calculation on the spin density of the one-electron oxidized form of all nine species led to the conclusion that the two species of the fully deprotonated SnTPyP ([SnTPyP(O^−^)_2_]^2−^) at pH > 6 and the singly deprotonated one ([SnTPyP(OH)(O^−^)]^−^ at pH 5–6 would have the most preferable reactivities against the two-electron oxidation of water-forming H_2_O_2_.

## Figures and Tables

**Table 1:** Summary of the molecular characteristics of SnTPyP as crucial data in designing MCSS.

SnTPyP	1		2		3		4		5		6		7		8		9	
p*K*_a_		0.7		1.4		2.4		3.0		3.9		4.3		5.0		5.9		
Fluorescence lifetime/ns	0.88		0.88		0.93		0.95		0.92		0.92		0.92		0.93		1.57	
Oxidation potential /Volt vs. SHE	-		1.79		*E_ox_* = −0.056 pH + 1.82 ^a^								1.58		1.55		1.54	
Spin density ^b^	0.001 0.001		0.002 0.007		0.011 0.011		0.012 0.012		0.011 0.011		0.010 0.010		0.010 0.010		0.993 0.002		0.988 0.001	
∆*E*(TiO_2_) ^c^ /eV (pH)	-		−0.11 (1.0)		−0.13 (2.0)		−0.13 (2.5)		−0.13 (3.5)		−0.13 (4.0)		−0.11 (4.5)		−0.09 (5.5)		−0.01 (7.0)	
∆*E*(SnO_2_) ^d^ /eV (pH)	-		−0.30 (1.0)		−0.32 (2.0)		−0.32 (2.5)		−0.32 (3.5)		−0.32 (4.0)		−0.30 (4.5)		−0.28 (5.5)		−0.20 (7.0)	

^a^ The oxidation potential estimated from the linear relation in the pH region (4.0–2.0) in the Pourbaix diagram (Figure 6b). ^b^ The spin densities on the two axial oxygen atoms calculated by DFT for each one-electron oxidized species of SnTPyP. ^c^ The energy difference for the electron injection from the excited SnTPyP into the conduction band of TiO_2_ at given pH conditions. The conduction band position of TiO_2_ (Volt vs. SHE) was calculated by −0.14 − 0.059 × (pH value). The excited energy of ^1^SnTPyP* (0-0 band) was taken as 2.1 eV. ^d^ The energy difference for the electron injection from the excited SnTPyP into the conduction band of SnO_2_ at given pH conditions. The conduction band position of SnO_2_ (Volt vs. SHE) was calculated by +0.05 − 0.059 × (pH value). The excited energy of ^1^SnTPyP* (0-0 band) was taken as 2.1 eV.

## Data Availability

The research data are available at Tokyo Metropolitan University Institutional Repository (https://irdb.nii.ac.jp/00891/0000797949, registered on 23 January 2017, disclosed on 15 August 2022).

## Supplementary Materials

The following supporting information can be downloaded at: https://www.mdpi.com/article/10.3390/molecules28041882/s1, SI-S1: Synthesis of SnTPyP; Figure S1: Fluorescence spectra of SnTPyP; Figure S2: ^1^H NMR of SnTTP and SnTMPyP; Figure S3: Change of the Electron densities represented as ∆(NBO charge) of the free pyridyl nitrogen atoms upon each single-protonation; Figure S4: Spin population of one-electron oxidized form of the nine species of SnTPyP.
