# Supplementary material for: Molecular Characteristics of Water-Insoluble Tin-Porphyrins for Designing the One-Photon-Induced Two-Electron Oxidation of Water in Artificial Photosynthesis
Source: Molecules. 2023 Feb 16;28(4):1882. doi: 10.3390/molecules28041882 (PMC9963784; doi:10.3390/molecules28041882)
Supplement: Supplementary file 1 [file molecules-28-01882-s001.zip › molecules-2200702-supplementary.pdf]

Supporting Materials

Molecular Characteristics of Water-Insoluble Tin-Porphyrins for Designing the One-Photon-Induced Two-Electron Oxidation of Water in Artificial Photosynthesis

Arun Thomas [1], Yutaka Ohsaki [2], Ryosuke Nakazato [2], Fazalurahman Kuttassery [3], Siby Mathew [2], Sebastian Nybin Remello [4], Hiroshi Tachibana [2] and Haruo Inoue [2,*]

[1] *Department of chemistry, St. Stephens College, Uzhavoor, P.O. 686634, Kottayam, India*
[2] *Department of Applied Chemistry, Graduate School of Urban Environmental Sciences, Tokyo Metropolitan University, 1-1 Minami-Osawa Hachioji, Tokyo 192-0397, Japan*
[3] *Department of Chemistry, University of Calicut, Thenhipallam P.O. 673635, Kerala, India*
[4] *Department of Applied Chemistry, Cochin University of Science and Technology, Kochi, P.O. 682022, Kerala, India*

List of Supporting Materials

S1 Synthesis of SnTPyP.

Fig. S1 Fluorescence spectra of SnTPyP.

Fig. S2 ^{1}H NMR of SnTTP and SnTMP.

Fig. S3 Change of the Electron densities represented as Δ(NBO charge) of the free pyridyl nitrogen atoms upon each single-protonation.

Fig. S4 Electron spin population of the one-electron oxidized form of SnTPyP.

S1. Synthesis of SnTPyP

Synthesis of *trans*-dihydroxy-5,10,15,20-tetra(4-pyridyl)porphyrinate tin (IV): [Sn(IV)TPyP(OH)$_2$]

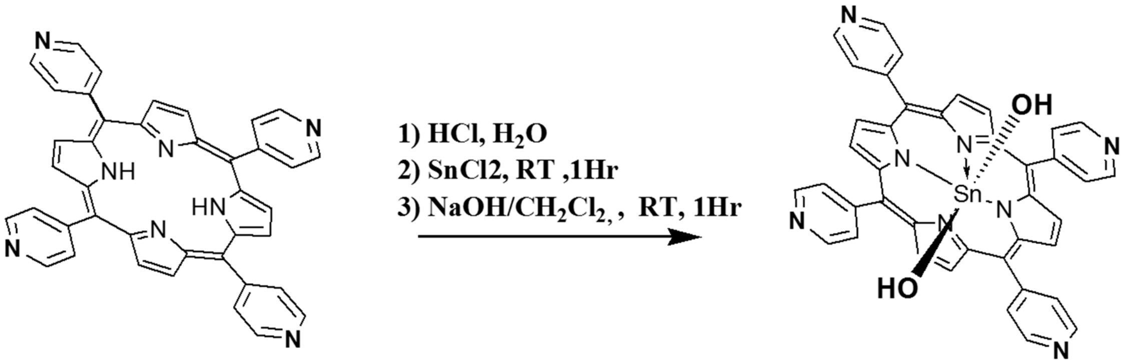

5,10,15,20-tetra(4-pyridyl) porphyrin H$_2$TPyP (25 mg: 0.041 mmol), which is not soluble in water, was made soluble in 10 mL water by adding HCl (pH was adjusted at 1.5) and the reaction mixture was kept stirring. SnCl$_2$ (30 mg: 0.13 mmol) was added to keep stirring for 1 hour. The progress of the reaction was monitored by UV Vis spectra. After the completion of the reaction, the reaction mixture was carefully neutralized by adding NaOH aqueous solution (1 M) to deprotonate the Sn(IV)TPy(4H$^+$)P into Sn(IV)TPyP. Then dichloromethane (DCM) was added to fully solubilize Sn(IV)TPyP in the DCM layer and was washed well with water by stirring. DCM layer was separated and cold hexane was added to precipitate violet colored crystals. Yield 93%.

EA: Obs C 59.72%, H 3.65%, N 13.87%, calcd for [Sn(IV)TPyP(OH)$_2$](H$_2$O)$_2$ C 59.65%, H 3.75%, N 13.93%. ^{1}H NMR (500 MHz, CDCl$_3$, TMS) δ = 9.16 (s, 8H, satellite $J_{Sn\text{-}H}$ 10 Hz), 8.28 (d, 8H, J = 5 Hz), 9.14 (d, 8H, J = 5 Hz), −7.46 (s(broad), axial OH). ^{13}C NMR (500 MHz, CDCl$_3$, TMS) δ = 118.9, 129.9, 133.0, 146.1, 148.76. ^{119}Sn NMR (500 MHz, D$_2$O, SnMe$_4$) δ −570.3 ppm (Supporting Information: SI-2 b)). ESI-MS: *m/z* = 768 as SnTPyP(O$^-$)$_2$. UV-Vis: ε = 6.36 × 10^5 M^{-1} dm^{-3} (λ_{max}: 418 nm in MeOH), 2.15 × 10^4 M^{-1} dm^{-3} (λ_{max}: 553 nm in MeOH).

Fig. S1.

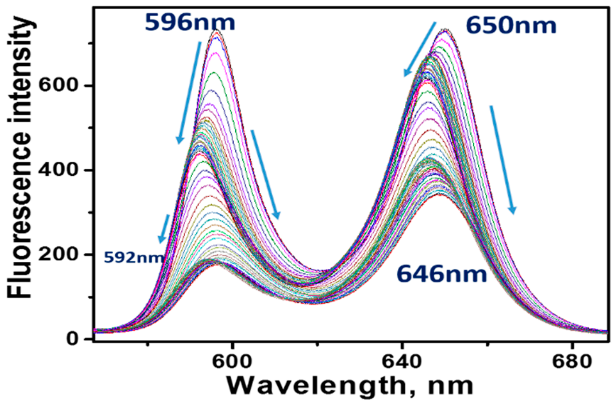

Fig. S1a. Fluorescence spectra of SnTPyP in CH_3CN/H_2O (8/2, *v/v*) excited at 420 nm under various pH conditions (pH 7 to 0.1).

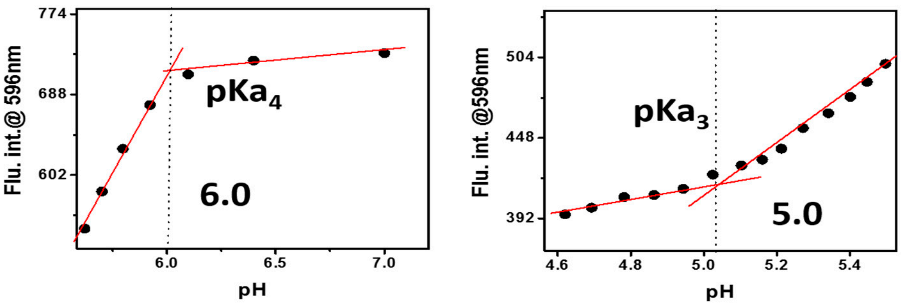

Fig. S1b. Plots of the fluorescence intensity of SnTPyP in CH_3CN/H_2O (8/2, *v/v*) against pH from 7 to 4.5.

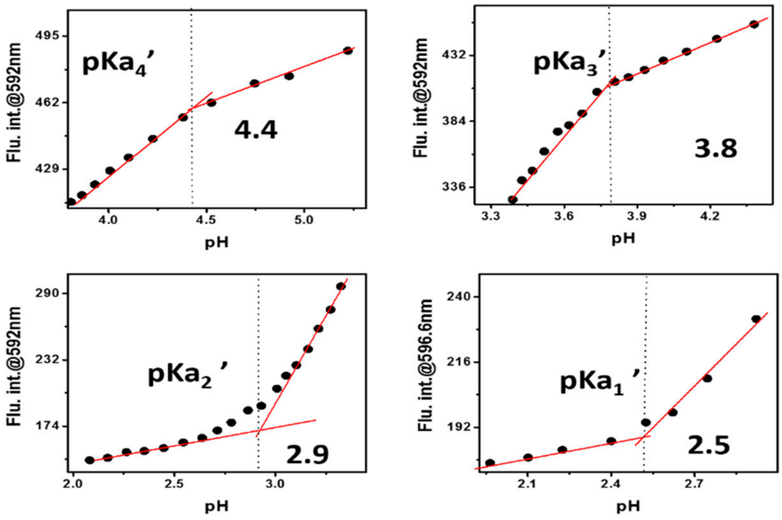

Fig. S1c. Plots of the fluorescence intensity of SnTPyP in CH_3CN/H_2O (8/2, *v/v*) against pH from 4.5 to 2.1.

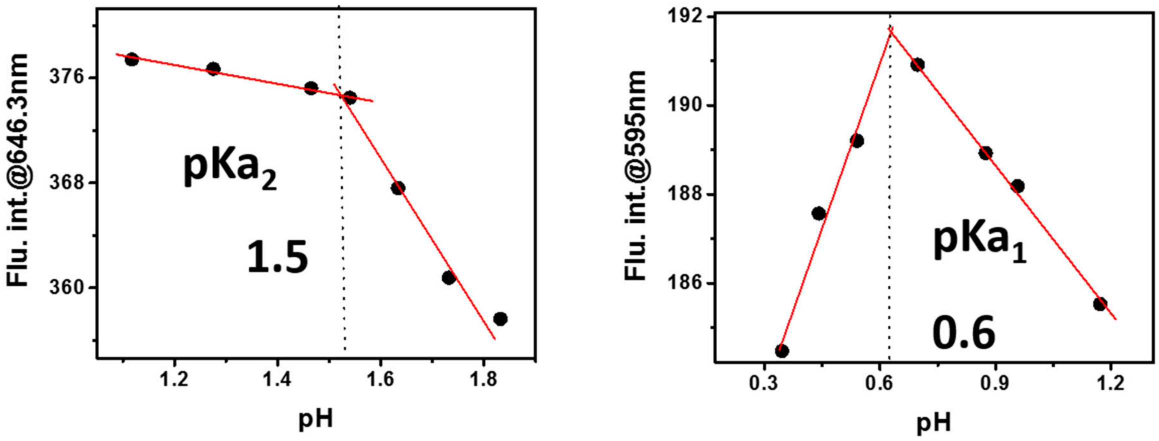

Fig. S1d. Plots of the fluorescence intensity of SnTPyP in CH_3CN/H_2O (8/2, *v/v*) against pH from 2.2 to 0.1.

Fig. S2.

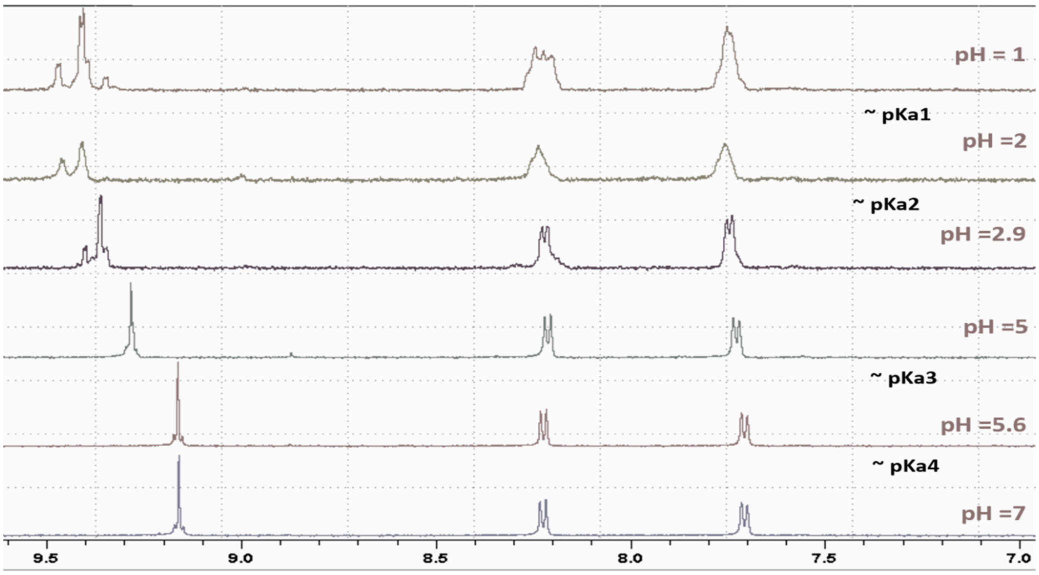

Fig. S2a. ^{1}H NMR of SnTTP in in CD_3CN/D_2O (8/2, *v/v*) at various pH near pKa.

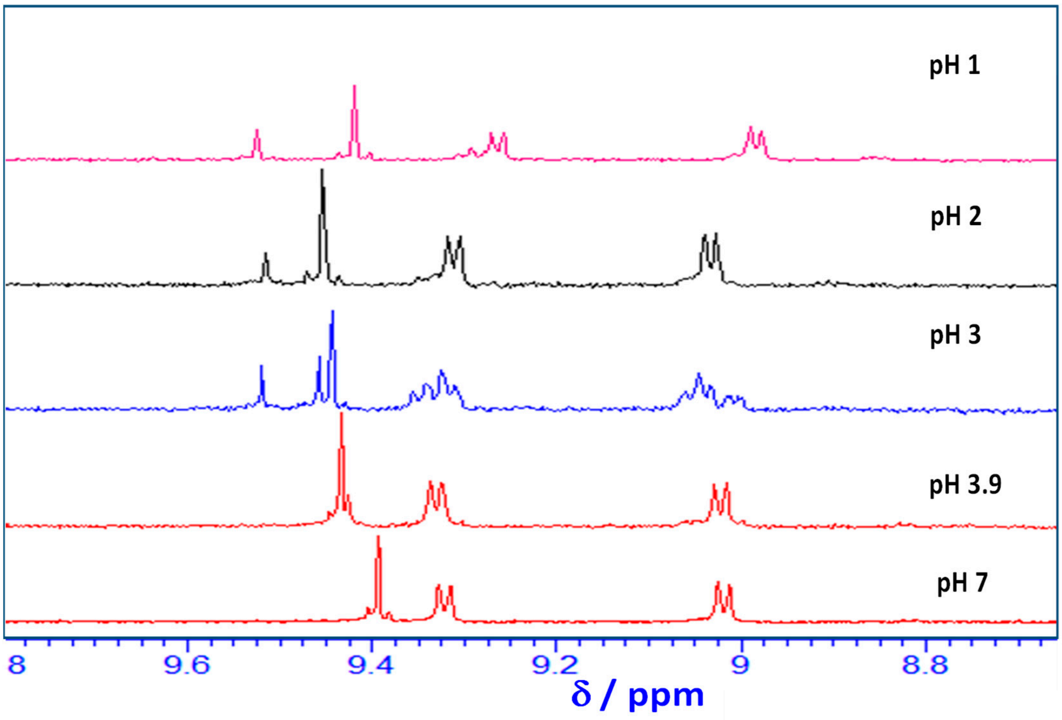

Fig. S2b. ^{1}H NMR of SnTMPyP in D_2O at various pH near pKa.

Fig. S3.

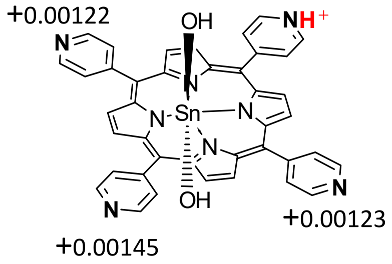

Fig. S3a. Change of the Electron densities represented as Δ(NBO charge) of the free pyridyl nitrogen atoms upon single-protonation.

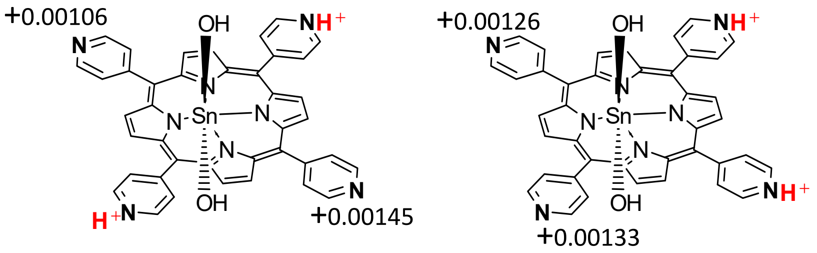

Fig. S3b. Change of the Electron densities represented as Δ(NBO charge) of the free pyridyl nitrogen atoms upon double-protonation either in *trans*- or *cis*-position.

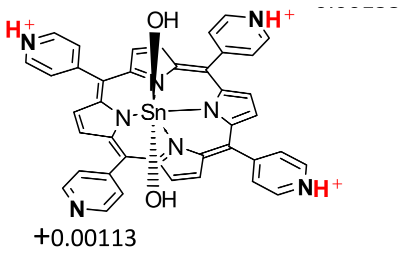

Fig. S3c. Change of the Electron densities represented as Δ(NBO charge) of the free pyridyl nitrogen atom upon triple-protonation.

Fig. S4.

Electron spin population of the one-electron oxidized form of the nine differently protolytic species of SnTPyP calculated by DFT (Gaussian16 UB3LYP/6-31G* SCRF(PCM: Water)), cutoff = 0.005.

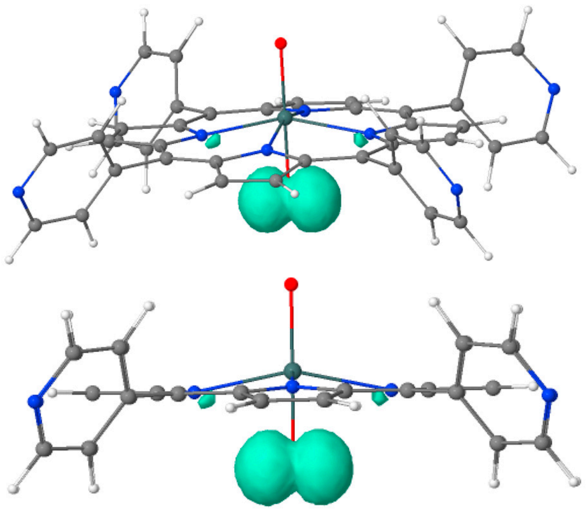

Fig. S4a. Electron spin population of $[SnTPyP(O^{-})(O)^{\bullet}]^{-\bullet}$ calculated by DFT.

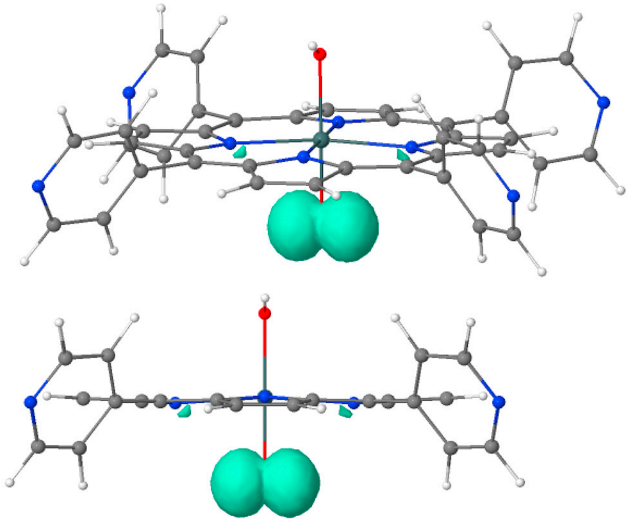

Fig. S4b. Electron spin population of $[SnTPyP(OH)(O^{\bullet})]^{\bullet}$ calculated by DFT.

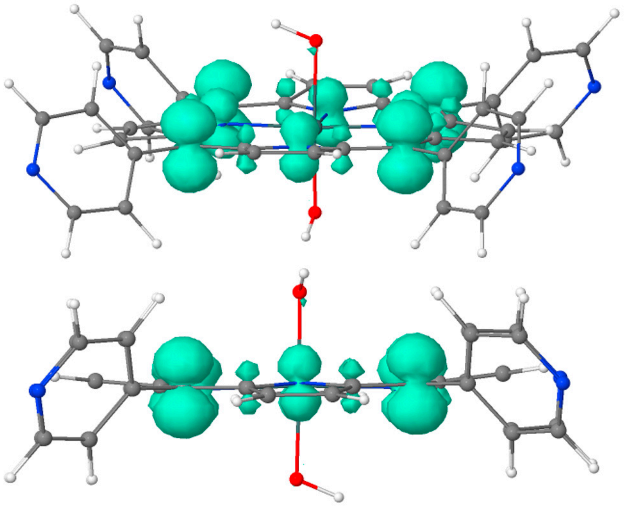

Fig. S4c. Electron spin population of $[SnTPyP(OH)_2]^{+\bullet}$ calculated by DFT.

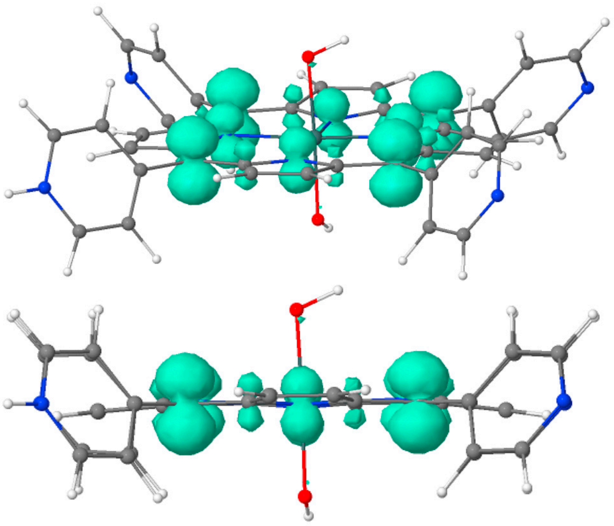

Fig. S4d. Electron spin population of $[SnT(PyH^{+})P(OH)_2]^{2+\bullet}$ calculated by DFT.

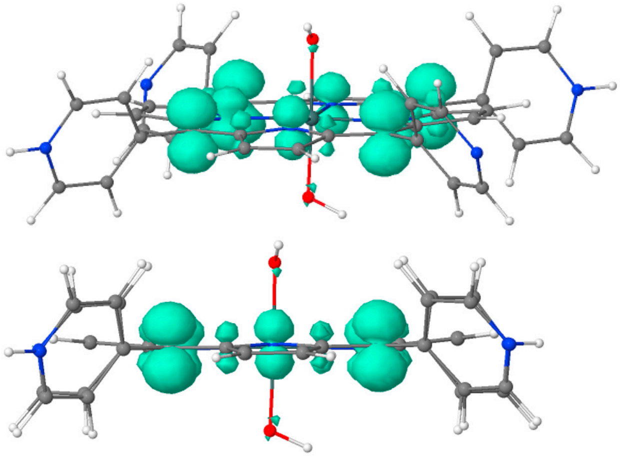

Fig. S4e. Electron spin population of $[SnTPy(PH_2^{2+})(OH)_2]^{3+\bullet}$ calculated by DFT.

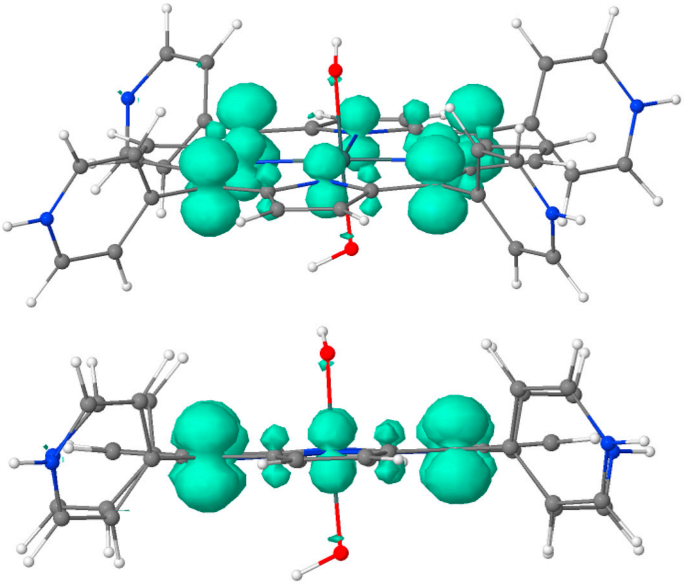

Fig. S4f. Electron spin population of $[SnTPy(PH_3^{3+})(OH)_2]^{4+\bullet}$ calculated by DFT.

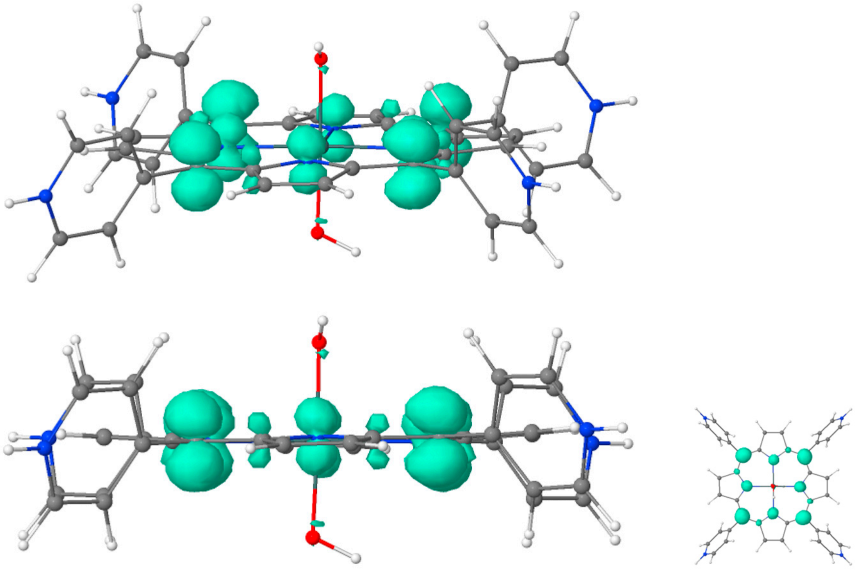

Fig. S4g. Electron spin population of [SnTPy(PH_4^{4+})$(OH)_2$]$^{5+\bullet}$ calculated by DFT.

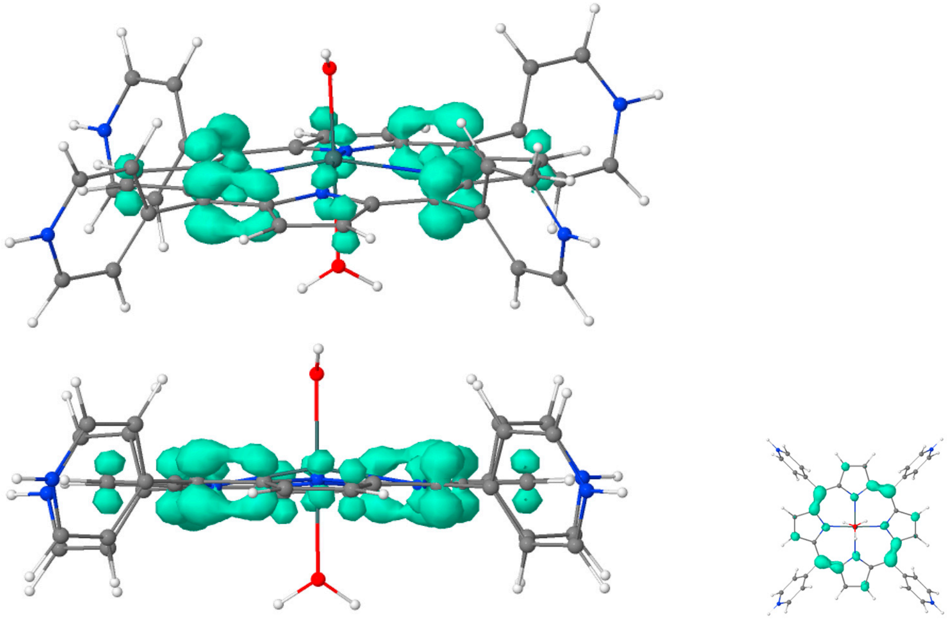

Fig. S4h. Electron spin population of [SnTPy(PH_4^{4+})(OH)(OH_2)]$^{6+\bullet}$ calculated by DFT.

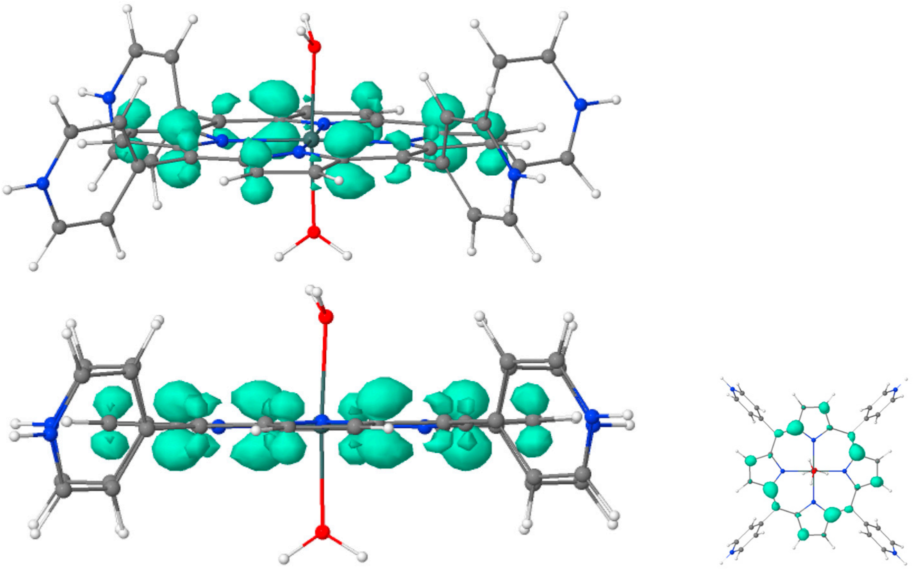

Fig. S4i. Electron spin population of $[SnTPy(PH_4^{4+})(OH_2)_2]^{7+\bullet}$ calculated by DFT.
